# Differential diagnosis between late-onset bipolar disorder vs. behavioral profile frontotemporal dementia: a diagnostic challenge

**DOI:** 10.1192/j.eurpsy.2025.1054

**Published:** 2025-08-26

**Authors:** E. Gil Benito, I. Álvarez Correa, A. Castelao Escobar, S. Castelao Almodovar, A. Pérez Balaguer, B. Gómez Esteban

**Affiliations:** 1 Psiquiatría, Hospital El Escorial, El Escorial; 2 Psiquiatría, Hospital Puerta de Hierro, Majadahonda, Spain

## Abstract

**Introduction:**

Maniform symptoms for the first time after the age of 50 are not common, although we can find it in our clinical practice due to the increasing aging of the world’s population.

**Objectives:**

We present a clinical case of a patient with manifest behavioral symptoms where a differential diagnosis is made between Late-onset bipolar disorder vs Frontotemporal dementia with a behavioral profile (FTD)

**Methods:**

62-year-old male with a history of type II diabetes, hypertension and L4-L5 disc herniation. Psychiatric history of recurrent depressive disorder and dysfunctional personality. Treated with venlafaxine 150 mg and gabapentin 600 mg DMD. He was on sick leave from his company due to lower back pain. Married with two children, dysfunctional relationship with them. He was admitted to Psychiatry for the first time in February 2024 due to behavioral disturbances of 5 days’ duration. He was verbose, irritable and described “being better than ever”. A few days earlier he took a Tadalafil tablet, an event that he related to the onset of the condition. Since then, there has been an increase in psychomotor activity, disinhibition and exalted mood. He reported having contact with high-ranking political figures. Upon discharge from hospital, he was diagnosed with an Unspecified Manic Episode and was prescribed Lithium 800mg DMD and Risperidone extended release 75 mg monthly. The symptoms did not improve, he abandoned the treatment and was admitted for the second time in April 2024 where Valproic 1000 mg DMD, Olanzapine 20mg DMD and Risperidone 6 DMD were prescribed.

**Results:**

He has a poor outpatient evolution with loss of autonomy, physical deterioration, hyperfamiliarity, behavioral disinhibition and no awareness of the disease. Given the suspicion of an organic condition, he was referred to Neurology consultations where a cranial magnetic resonance imaging was performed. with results of punctate and hyperintense images in T2 localized in subcortical white matter of nonspecific character and Mini-ACEII test: 22/30. Waiting for PET-CT and with a diagnosis of possible Frontal Release Syndrome to rule out behavioral variant FTD.

**Conclusions:**

Within the differential diagnosis of the condition we find Late-onset bipolar affective disorder and behavioral variant FTD. The first presents with inappropriate, repetitive and stereotyped behavior, as well as a progressive and gradual deterioration. While late bipolar disorder presents with self-limiting episodes and more manifest symptoms. In a PET-CT suggestive of FTD it is likely to find areas of hypoperfusion in frontal and temporal regions. The differential diagnosis between both is a challenge in clinical practice.

**Disclosure of Interest:**

None Declared

